# ACTN4 Promotes the Proliferation, Migration, Metastasis of Osteosarcoma and Enhances its Invasive Ability through the NF-κB Pathway

**DOI:** 10.1007/s12253-019-00637-w

**Published:** 2019-03-16

**Authors:** Qingshan Huang, Xiaodong Li, Zhen Huang, Fengqiang Yu, Xinwen Wang, Shenglin Wang, Zhizhen He, Jianhua Lin

**Affiliations:** Department of Orthopedics, The First Affiliated Hospital of Fujian Medical University, Fuzhou, 350005 China

**Keywords:** Osteosarcoma, ACTN4, Invasion, Metastasis, NF-κB

## Abstract

Alpha-actinin-4 (ACTN4) is associated with different types of tumors, but its role in osteosarcoma (OS) is not known. We aimed to investigate the effect of ACTN4 on the growth, migration, invasion and metastasis of OS. We further explored the possible mechanism of how ACTN4 affects the development of OS. First, the expression of ACTN4 in OS tissues and OS cell lines was analyzed by PCR. Second, the role of ACTN4 in the development of OS was explored by the proliferation, scratch, and invasion assays. We further explored the effect of ACTN4 on OS growth in an orthotopic xenograft model of nude mice. In addition, we used hematoxylin and eosin (HE) staining of lung tissues in nude mice to observe the effect of ACTN4 on lung metastasis of OS. Finally, rescue experiments further investigated the role of NF-κB on ACTN4 in the development of OS. ACTN4 was highly expressed in OS tissues and OS cell lines. In vitro experiments demonstrated that reducing ACTN4 expression inhibited the proliferation, migration, and invasion of OS. In contrast, overexpression of ACTN4 promotes these effects. In vivo experiments further validated that ACTN4 promoted the growth of OS. The HE staining of lungs in nude mice revealed that ACTN4 promoted lung metastasis of OS. In addition, we found that ACTN4 enhanced the ability of OS to invade, through the NF-κB pathway. ACTN4 promotes the proliferation, migration, metastasis of OS and enhances its invasion ability through the NF-κB pathway.

## Introduction

Osteosarcoma (OS) is a prevalent primary malignant bone tumor [1] predominantly affects children and adolescents [2]. OS typically occurs in the metaphysis of long bones [3] and has a high incidence of lung metastasis and local aggressiveness [4]. The survival rate of OS is 60–70% after surgery and neoadjuvant chemotherapy [5, 6]. However, no further increase in survival rates has been observed over the past 30 years [5, 7]. Metastasis and recurrence are the most difficult problems in the treatment of OS. The 5-year survival rate is only about 20% [8]. Therefore, it is imperative to understand the underlying molecular mechanisms involved in the invasion and metastasis of OS. New therapeutic targets need to be discovered to reduce the recurrence and metastasis of OS and improve the survival rate of patients.

Alpha-actinin-4 (ACTN4) is an actin-binding protein that belongs to the spectrin superfamily [9]. It has four isoforms: ACTN1, ACTN2, ACTN3 and ACTN4. ACTN2 and ACTN3 are expressed in muscles, while ACTN1 and ACTN4 are ubiquitously expressed in non-muscle cells [10, 11]. ACTN4 has many roles in non-muscle cells, including cellular motility and cell adhesion [12, 13]. In gastric cancer cells, knockdown of ACTN4 increases cell adhesion and reduces migration and invasion of cells [14]. Overexpression of ACTN4 leads to disease development and a poor prognosis [15]. However, the role of ACTN4 in OS is rarely reported. Our previous study showed that ACTN4 is highly expressed in patients with OS metastasis [16]. We wanted to investigate the effect of ACTN4 on the proliferation, migration, invasion and metastasis of OS.

Nuclear factor-κB (NF-κB) is a protein complex, which controls the transcription of various target genes involved in cell proliferation, apoptosis and metabolism [17]. The NF-κB family consists of the subunits p50, p52, p65 (RelA), c-Rel and Rel B, and form a variety of heterodimers with distinct functions [18]. Of these, the p50/65 heterodimer is the most widely distributed, and is found in almost all cell types. After phosphorylation, p65 binds to p50 to form a heterodimer and is transferred to the nucleus to initiate downstream gene transcription. Studies have shown that aberrantly activated p65 (RelA) contributes to tumor development and progression [19, 20]. In addition, the existence of co-immunoprecipitation between p65 and ACTN4 in non-small lung carcinoma cells has been reported [21]. Our study will further verify whether ACTN4 affects the progression of OS through the NF-κB pathway.

In this study, we transfected OS cells with lentivirus vectors to either reduce or overexpress the ACTN4 gene. We then observed the effects of ACTN4 expression on the proliferation, migration and invasion of OS cells, and the effects of ACTN4 on the growth and metastasis of OS were further validated in nude mice. In addition, we conducted a preliminary study on the mechanism of ACTN4 affecting the invasion of OS.

## Materials and Methods

### Cell Culture

The human OS cell lines hFOB 1.19, U_2_OS and MNNG/HOS (HOS) were obtained from the Typical Culture Preservation Committee of the Chinese Academy of Sciences, Shanghai, China. Cells were maintained in DMEM medium (Hyclone, Logan, UT, USA) supplemented with 10% fetal bovine serum (Gibco, Gaithersburg, MD, USA) and 1% penicillin-streptomycin (Hyclone, Logan, UT, USA) in an atmosphere of 5% CO_2_ at 37 °C.

### Tissue Specimens

Twenty OS tissues and their matched adjacent noncancerous tissues were collected at the department of Bone tumor, First Affiliated Hospital of Fujian Medical University, China. All tissues were stored at −80 °C carefully after surgery until RNA isolation. All cases were diagnosed as OS by pathology.

### Reverse Transcription-Quantitative Polymerase Chain Reaction (RT-qPCR)

Total RNA from tissues and cells were extracted using the TRIzol reagent (Invitrogen) according to manufacturer’s instructions. First, The RNA sample was synthesized into cDNA by M-MLV Reverse Transcription kit (Promega, Madison, WI, USA). Then it was amplifed by qPCR with an ABI 7900HT Real-time PCR system. The mRNA of the housekeeping gene GAPDH was selected as an internal reference.

### ACTN4 Knockdown and Overexpression Cell Lines

The knockdown (sequences:5’-GCACCAACCTGAACAATGCCTTCGAA-3′) and overexpression lentivirus vectors, and the corresponding negative control vectors were purchased from Hanbio Biotechnology Co. Ltd. (Shanghai, China). U_2_OS and HOS cells were infected with lentiviral particles according to the manufacturer’s instructions. The lentiviral vectors contained EGFP. Thus, the cells that were successfully infected with lentiviruses showed green fluorescence. Each transfected cell line was divided into four groups: sh-ACTN4 (transfected with knockdown lentivirus), NC1 (transfected with control lentivirus of sh-ACTN4), oe-ACTN4 (transfected with overexpressed lentivirus) and NC2 (transfected with control lentivirus of oe-ACTN4).

### Western Blot Analysis

Cells were washed with PBS and lysed in RIPA buffer containing a protease inhibitor cocktail (Beyotime, Shanghai, China). The total cellular protein was separated on an 8% SDS polyacrylamide gel. The protein was then transferred onto PVDF membranes (Millipore, Bedford, MA, USA). The PVDF membranes were incubated in 5% skim milk for 2 h. The membranes were incubated overnight at 4 °C with primary antibodies against GAPDH (Abcam, Cambridge, MA, USA), ACTN1–4 (Abcam, Cambridge, MA, USA), P65 (Abcam, Cambridge, MA, USA), and p-P65 (Abcam, Cambridge, MA, USA). Membranes were then incubated with the appropriate secondary antibody for 2 h at room temperature. Finally, the protein bands were detected using a chemiluminescence detection system (Amersham Biosciences, Piscataway, NJ, USA).

### CCK-8 or EDU for Cell Proliferation Assay

Transfected cells were plated in 96-well culture dishes with a density of 3000 cells/well. Cells were incubated for 0 h, 24 h, 48 h, 72 h and 96 h, and then treated with 10 μl Cell Counting Kit-8 (CCK-8) solution (Beyotime, Shanghai, China). An automated absorbance reader (Tecan Benelux BVBA, Mechelen, Belgium) was applied to measure the OD values at 450 nm.

The transfected cells were seeded into 6-well plates. EDU (5-ethynyl-2′ –deoxyuridine, Beyotime, Shanghai, China) was added after 12 h with a final concentration of 10 μM. These cells were incubated at 37 °C for 2 h. The medium was then removed, and 1 ml of 4% paraformaldehyde was added to each well for fixation, and the cells were washed 3 times with the washing solution. Then, 1 ml of permeabilization solution was added per well and incubated at room temperature for 10–15 min. The permeabilization solution was removed and the cells were washed 3 times with washing solution. Finally, the nuclei were stained with DAPI (Beyotime, Shanghai, China) and photographs were taken under a fluorescence microscope.

### Wound Healing Assay

Cells were seeded into 6-well plates. A confluent cell monolayer was used to make scratches with a sterile pipette tip. Floating cells were washed twice with PBS. The plates were then maintained at 37 °C. The microscope was used to observe scratch changes every 12 h. The software, Image-Pro Plus 6.0 (Media Cybernetics, USA) was used to calculate the area of the scratches.

### Transwell Invasion Assay

The invasion assay was estimated by 24-well transwell chambers (Corning Inc., Corning, NY, USA). The matrigel (BD Biosciences, Franklin Lakes, NJ, USA) was inserted into the upper chamber with an 8 μm pore size. Cells (1 × 10^5^ cells / well) were seeded into the upper chamber with serum-free medium and the lower chambers were filled with 700 μl of complete medium. After 24 h of incubation at 37 °C, the cells that remained on the upper side of the membrane were removed, and the cells that had migrated to the lower side were treated with paraformaldehyde and stained with crystal violent (Beyotime, Shanghai, China). All cells that migrated to the lower surface were counted.

### Animal Experiments

Male nude mice at 4 weeks old were purchased from SLAC, Shanghai, China. We anesthetized the mice and carefully rotated the cortical bone of the tibia to the proximal marrow cavity using a 27G syringe needle [22]. Then, 20 μl of cell suspension was slowly injected into the bone marrow cavity. The tumor size was measured every week, after 2 weeks of cell injection and the volume was calculated by the formula V = (length x width ^2^)/2 [23]. Six weeks after the cell injections, we performed cervical dislocation to kill the nude mice, and removed their tumors to perform immunofluorescence experiments. At the same time, we took out the lungs of nude mice for hematoxylin and eosin (HE) staining and counted the number of nodules in lung metastases.

### Immunofluorescence (IF)

The slices were thawed at room temperature, and then fixed with acetone at −20 °C for 5 min. After washing 3 times with PBS solution, the slides were permeabilized with 1% Triton X-100 for 15 min. The slides were washed again with PBS and blocked with 5% BSA at room temperature for 45 min. These were then incubated overnight with ACTN4 primary antibody (1:100, Abcam, Cambridge, MA, USA). After washes with PBST, the secondary antibody (Cy3-conjugated goat anti-rabbit IgG, Servicebio, Wuhan, Hubei, China) was incubated for 2 h at room temperature. The nuclei were finally stained with DAPI.

### Statistical Analysis

All experiments were repeated at least 3 times, and data were expressed as the mean ± SD. The two-sample mean comparison was performed using a group t-test, unless otherwise specified. SPSS 19.0 software was used for data analysis. *P* < 0.05 was considered statistically significant.

## Results

### Expression of ACTN1–4 in HOS and U_2_OS Cell Lines

**Fig. 1:**
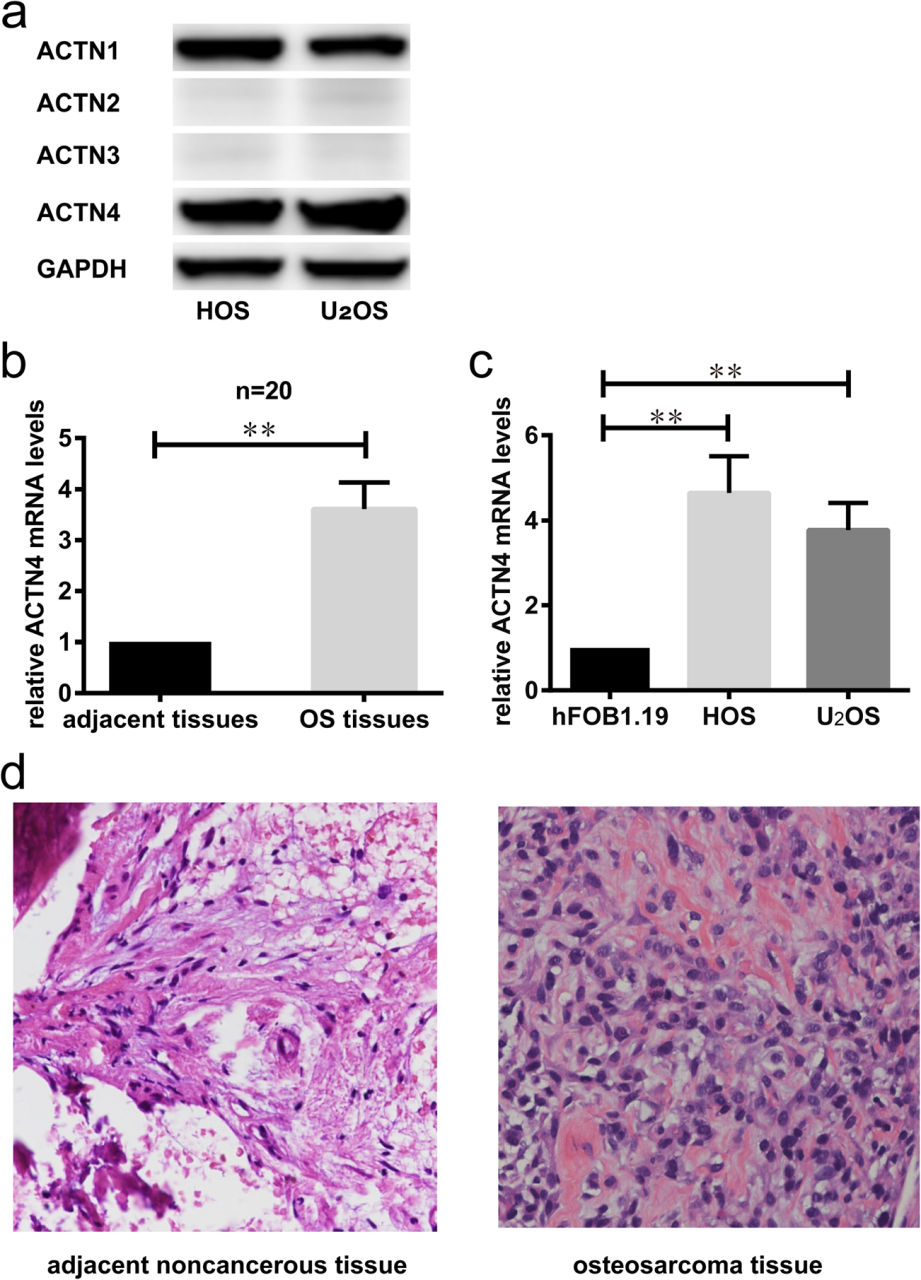
**a** The expression of ACTN1–4 in HOS and U_2_OS cell lines was analyzed by western blot. **b.** The mRNA level of ACTN4 were determined by RT-qPCR in 20 OS tissues compared with adjacent noncancerous tissues. **c.** The mRNA level of ACTN4 were determined by RT-qPCR in OS cell lines (HOS /U2OS) and osteoblastic cell line (hFOB1.19). **d.** The cases were diagnosed as OS by pathology. A small amount of chronic inflammatory cell infiltration and fibrosis can be seen in the pathological results of the adjacent noncancerous tissues. In osteosarcoma tissue, the morphology and staining of cells and nuclei are associated with the diagnosis of OS. Data are shown as means ± SD. **P < 0.05

We examined the expression of ACTN1–4 in HOS and U_2_OS cell lines by Western blot. It can be observed that ACTN1 and ACTN4 are abundantly expressed in these two cell lines. However, ACTN2 and ACTN3 were hardly expressed in both cell lines. Further, we can find that the expression level of ACTN4 is slightly higher than that of ACTN1 (Fig. 1a).

### ACTN4 Is Up-Regulated in OS Specimens and Cell Lines

We examined the expression of ACTN4 in cancer tissues and adjacent non-tumor tissues in OS cases by RT-qPCR. The clinical and pathological data of 20 cases in the study are summarized in the table (Table 1). The results showed that the expression of ACTN4 was up-regulated in OS tissues, which was significantly higher than that in adjacent tissues (Fig. 1b). Furthermore, we also compared the expression of ACTN4 in OS cell lines (HOS /U2OS) and osteoblastic cell line (hFOB1.19), and we can also find that ACTN4 is up-regulated in OS cell lines (Fig. 1c). In addition, all cases were diagnosed as OS by pathology (Fig. 1d).

**Table 1:** The clinical and pathological data of 20 cases

Case	Gender	Age(years)	Site	Metastasis	Enneking staging	Histopath type
1	F	16	Humerus	Y	III	O
2	M	46	Ribs	Y	III	C
3	F	9	Femur	N	II	O
4	F	11	Ribs	N	II	O
5	M	20	Femur	N	II	O
6	F	13	Humerus	N	II	Fi
7	M	50	Humerus	N	II	C
8	F	64	Femur	Y	III	O
9	F	11	Femur	N	II	O
10	F	13	Femur	Y	III	O
11	M	61	Pelvis	N	II	C
12	M	15	Fibula	N	II	O
13	M	14	Tibia	N	II	O
14	F	13	Femur	N	II	O
15	M	9	Fibula	N	I	O
16	F	46	Femur	Y	III	C
17	F	26	Tibia	N	II	T
18	M	24	Femur	Y	III	O
19	M	11	Humerus	N	I	O
20	M	50	Humerus	N	II	O

*F* Female, *M* Male, *Y* Yes, *N* No, *O* Osteoblastic OS, *C* Chondroblastic OS, *Fi* Fibroblastic OS; *T* Telangiectatic OS

### Knockdown and Overexpression of ACTN4 Gene

**Fig. 2:**
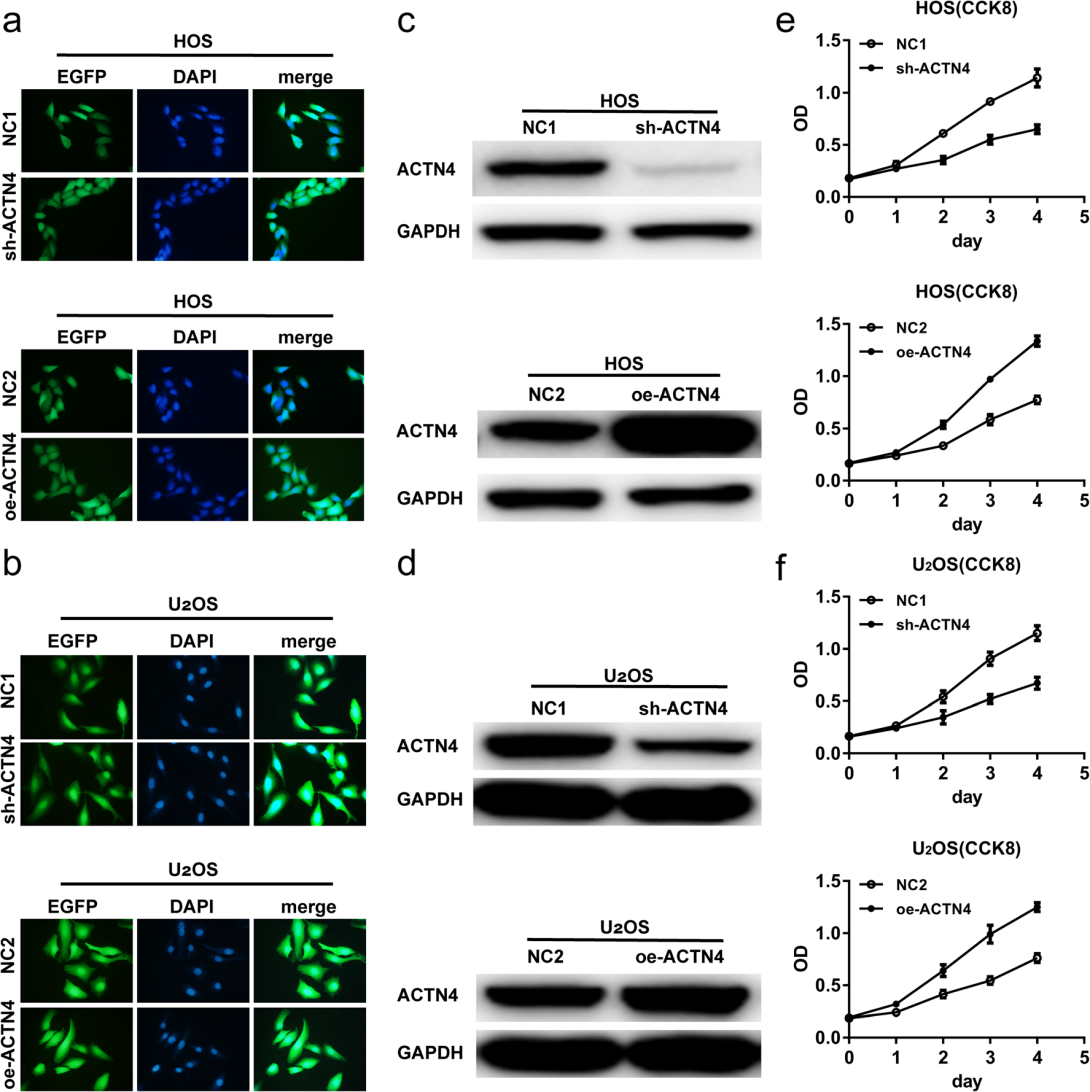
Interference and overexpression of ACTN4 gene in OS cells. Altering the expression levels of ACTN4 affect the proliferation of OS cells (CCK8). **a**, **b.** Stably transfected OS HOS cells or U_2_OS cells. **c**, **d.** Western blots to verify the effect of interference or overexpression of ACTN4 gene in HOS cells and U_2_OS cells. **e**, **f.** The effect of ACTN4 gene interference or overexpression on proliferation of HOS and U_2_OS cells detected by CCK8. Data are shown as means ± SD

The ACTN4 gene is ubiquitously expressed in both normal and tumor cells. Similarly, both the human OS cell lines HOS and U_2_OS express this gene. We successfully transfected these cell lines with lentiviral vectors with a transfection efficiency above 90% (Fig. 2a, b). To further verify the knockdown and overexpression of the ACTN4 gene, we used Western blot to detect the expression levels of ACTN4. The results showed that the expression levels of ACTN4 protein in the group sh-ACTN4 was significantly decreased, and the expression levels of the ACTN4 protein in the group oe-ACTN4 was significantly increased (Fig. 2c, d). Therefore, we successfully generated OS cells with different expression levels of ACTN4.

### Altering the Expression Levels of ACTN4 Affects the Proliferation of OS Cells

Cell proliferation is an important process in the development of OS. We used CCK8 to detect the proliferation of OS cells. We found that the proliferation of HOS cells was slower after ACTN4 gene expression was reduced (Fig. 2e). In contrast, the HOS cells increased their proliferative capacity after overexpression of the ACTN4 gene (Fig. 2e). We observed a similar trend in U_2_OS cells (Fig. 2f). We observed that the difference was even more pronounced, after the second day.

**Fig. 3:**
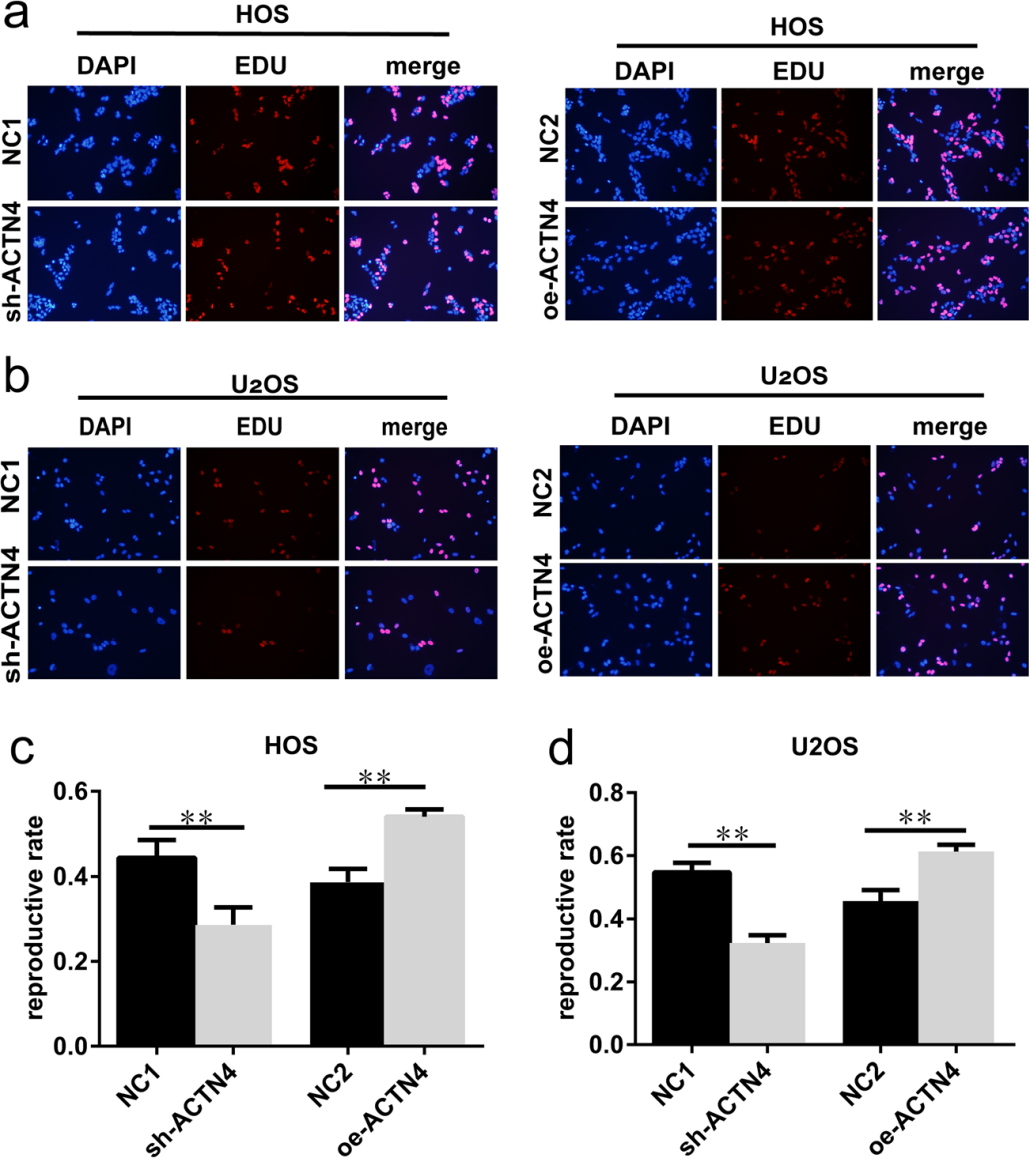
Altering the expression levels of ACTN4 affect the proliferation of OS cells (EDU). **a**, **c.** Detection of the effect of ACTN4 gene interference or overexpression on proliferation of HOS cells using the EDU kit. **b**, **d**. The effect of ACTN4 gene interference or overexpression on the proliferation of U_2_OS cells was measured using the EDU kit. Red shows cells in a proliferative state. Blue shows all the cells in the field of vision. Data are shown as means ± SD. **P < 0.05

We used the EDU kit to further verify the effect of ACTN4 expression changes on OS cells. We observed that all cells were in the proliferative phase. In HOS cells, we found that the proportion of cell proliferation was lower in the sh-ACTN4 group than in the NC1 group (Fig. 3a, c). In contrast, these cells showed enhanced proliferative capacity after overexpression of the ACTN4 gene (Fig. 3a, c). We observed similar results in U_2_OS cells (Fig. 3b, d). Therefore, we conclude that reducing the expression of ACTN4 inhibits the proliferation of OS cells, while overexpressing ACTN4 enhances the proliferation of OS cells.

### Altering the Expression Levels of ACTN4 Affects the Migratory Ability of OS Cells

**Fig. 4:**
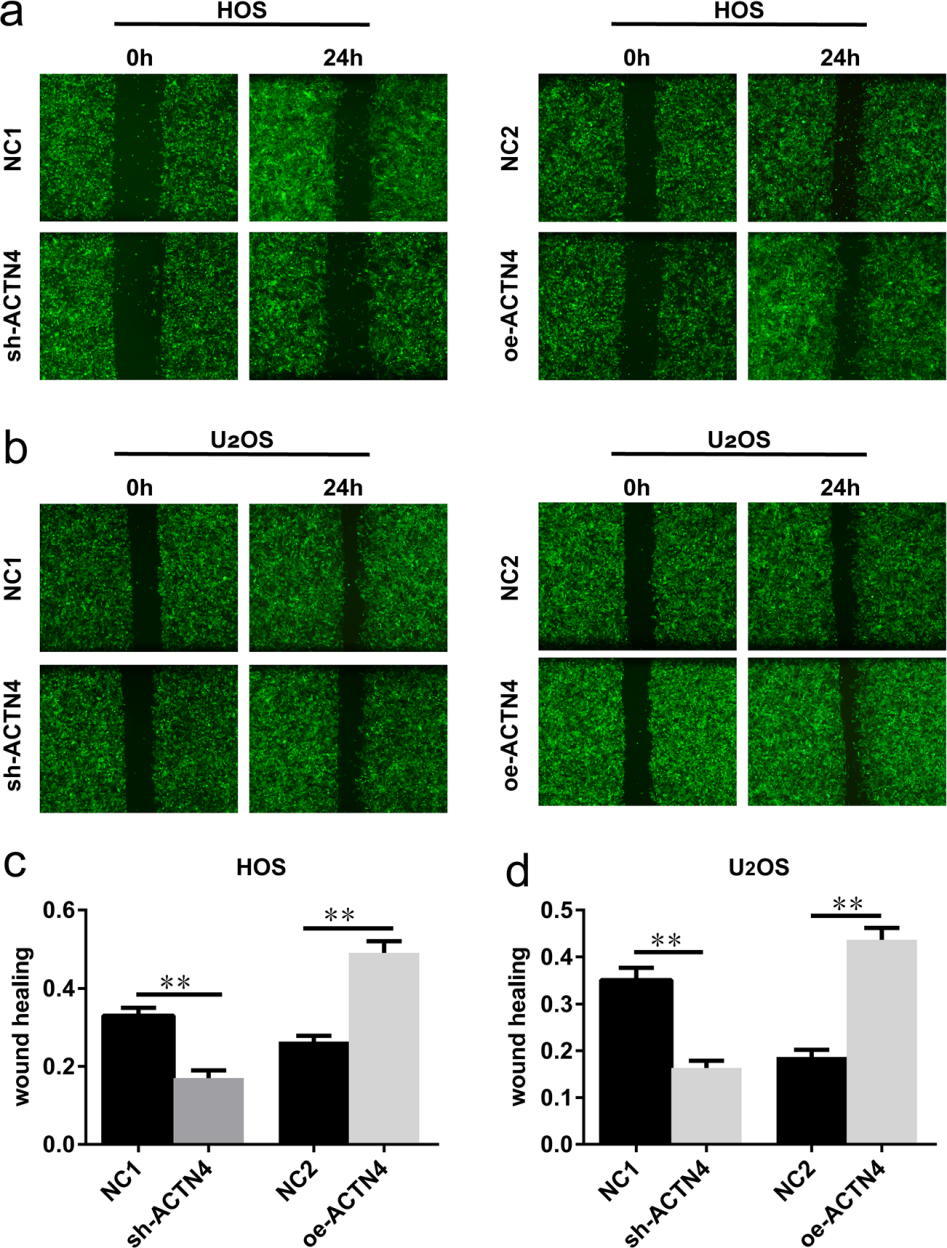
Altering the expression levels of ACTN4 affect the migratory ability of OS cells. **a**, **c.** Scratch test was used to verify the effect of different expression levels of ACTN4 on the migratory ability of HOS cells. **b**, **d.** Scratch experiments were used to verify the effect of different expression levels of ACTN4 on U2OS cell migration. Photographs were taken using a fluorescence microscope and their migratory ability was compared by calculating the rate of change of the scratch area. Data are shown as means ± SD. **P < 0.05

We used scratch experiments to detect the ability of OS cells to migrate. Since the vector contains the EGFP gene, we were able to observe the scratches with time using a fluorescence microscope. In HOS cells, the scratch healing rate of the sh-ACTN4 group was significantly slower than that of the NC1 group (Fig. 4a, c). The scratch healing ability of the oe-ACTN4 group was faster than that of the NC2 group (Fig. 4a, c). We observed a similar trend in U_2_OS cells (Fig. 4b, d). Our results show that decreasing the expression level of ACTN4 inhibits the migratory ability of OS cells. Conversely, increasing the expression levels of ACTN4 promotes the migratory ability of OS cells.

### Altering the Expression Levels of ACTN4 Affects the Invasive Ability of OS Cells

**Fig. 5:**
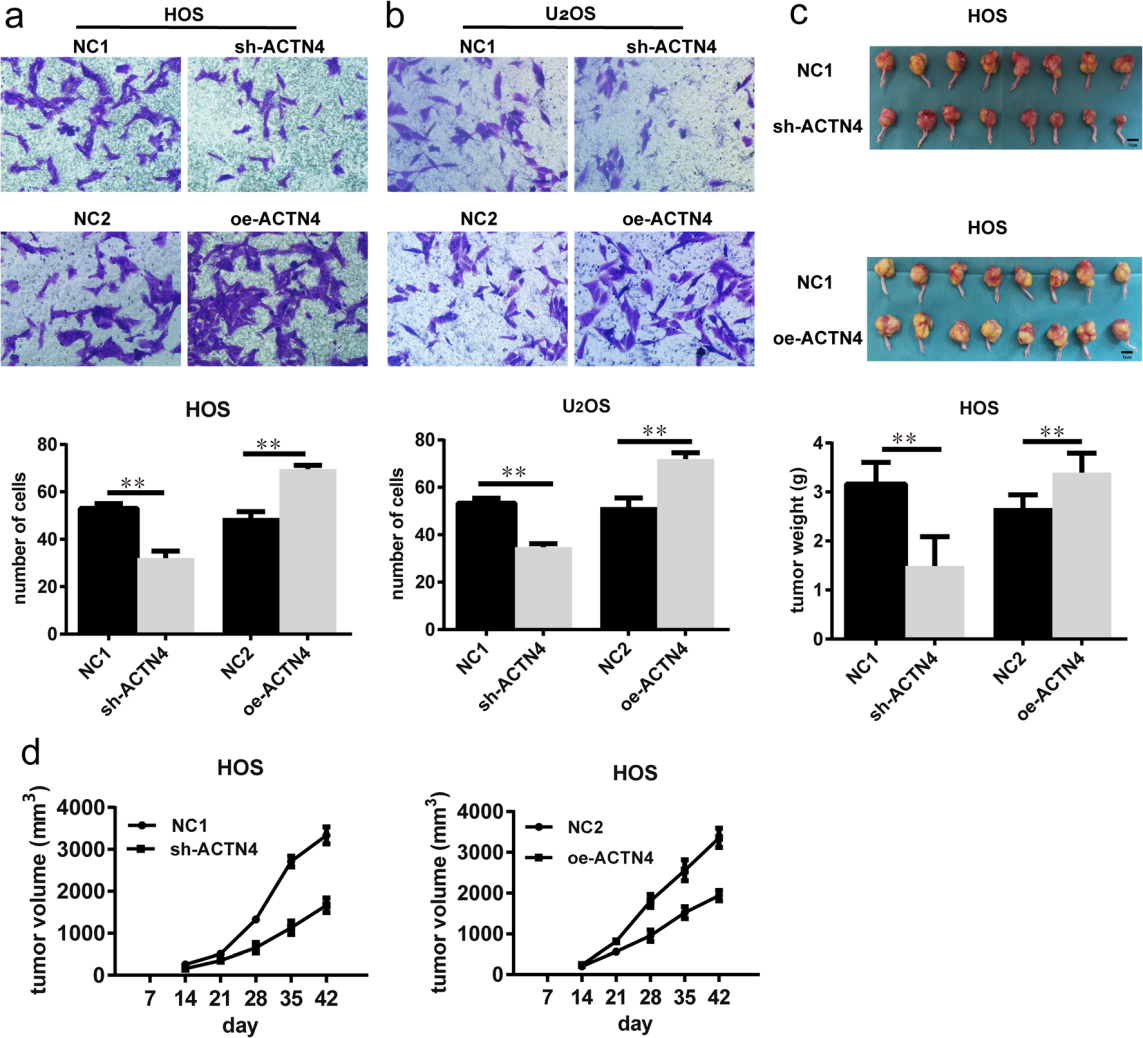
Altering the expression levels of ACTN4 affects the invasive ability of OS cells. Altering the expression levels of ACTN4 affect the growth of OS in vivo . **a**, **b.** Effects of different expression levels of ACTN4 on the invasive ability of HOS and U_2_OS cells. **c.** Growth of transplanted tumors in nude mice after 6 weeks of HOS cell injection. Bar, 1 cm. **d.** The size of the tumor is measured every week from the second week after cell injection. Tumor volume was calculated by the formula V = (length x width^2^)/2. Data are shown as means ± SD. **P < 0.05

The ability to invade is an important indicator of tumor malignancy. We simulated the invasion environment of OS using transwell chambers and matrigel. The invasive ability was compared by counting the number of OS cells crossing the chamber and matrigel. In HOS cells, we found that the number of cells in the sh-ACTN4 group was significantly less than the NC1 group (Fig. 5a). The number of cells in the oe-ACTN4 group was significantly more than that in the NC2 group (Fig. 5a). We observed a similar pattern in the U_2_OS cells (Fig. 5b). Our data shows that decreased ACTN4 expression inhibits the invasive ability of OS, and increased ACTN4 expression enhances the invasive ability of OS.

### Altering the Expression Levels of ACTN4 Affects the Growth of OS In Vivo

In vitro, we observed that the levels of ACTN4 expression affects the proliferation of OS. We wanted to investigate if this occurs in vivo, as well. Therefore, we transplanted HOS cells into the bone marrow cavity of the tibia of nude mice. From the second week onwards, we measured the size of the tumor once a week and plotted the growth curve (Fig. 5d). We observed that the growth rate of tumors in the sh-ACTN4 group was slower than that of the NC1 group, and the oe-ACTN4 group had a faster growth rate than the NC2 group. Six weeks after cell injection, we sacrificed the nude mice with cervical dislocation and removed the tumor tissues (Fig. 5c). We weighed the tumor tissues and obtained the average weight of each group of tumors (Fig. 5c). We found that the average tumor weight of the sh-ACTN4 group was smaller than that of the NC1 group. In contrast, the average tumor weight of the oe-ACTN4 group was greater than that of the NC2 group.

### Altering the Expression Levels of ACTN4 Affects the Metastasis of OS In Vivo

**Fig. 6:**
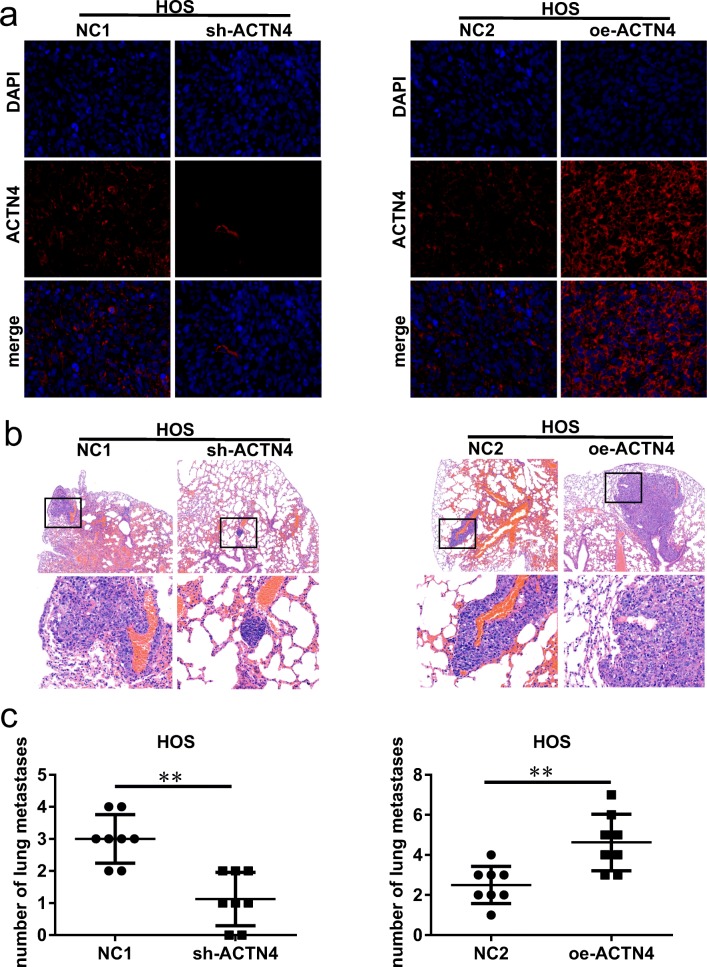
Altering the expression levels of ACTN4 affect the metastasis of OS in vivo. **a**. Immunofluorescence assay was used to detect the expression levels of ACTN4 in nude mice. **b.** HE staining of lung tissue after 6 weeks of cell injection. **c.** The number of lung metastases in nude mice after 6 weeks of cell injection (each lung tissue was randomly selected for 5 sections). Data are shown as means ± SD. **P < 0.05

We successfully reduced and overexpressed the ACTN4 gene in HOS cells, in vitro. We next asked if HOS cells could maintain the original expression levels of ACTN4 gene after inoculation into nude mice. For this purpose, we performed immunofluorescence experiments on the excised tumor tissues (Fig. 6a). We found that the ACTN4 levels in the sh-ACTN4 group were still lower than the NC1 group, while the ACTN4 levels in the oe-ACTN4 group were still higher than in the NC2 group. Therefore, we found no significant changes in the expression of ACTN4 after HOS cells were transplanted into nude mice. These data validate results from our animal experiments.

One of the challenges in OS treatment is tumor metastasis, and lungs are the most common site of OS metastasis [24]. Therefore, we wanted to explore if changes in the expression of ACTN4 affects the lung metastasis of OS. We performed HE staining after taking out the lung tissues in nude mice (Fig. 6b). The number of metastatic nodules in the lungs were counted (Fig. 6c). We found that there were fewer metastases in the sh-ACTN4 group than in the NC1 group, and the diameter of the metastases were smaller than in the NC1 group. In contrast, we observed the opposite result in the oe-ACTN4 group. Our data suggests that low levels of ACTN4 inhibit lung metastasis of OS, and high levels of ACTN4 promote lung metastasis of OS.

### ACTN4 Affects the Invasive Ability of OS through the NF-κB Pathway

**Fig. 7:**
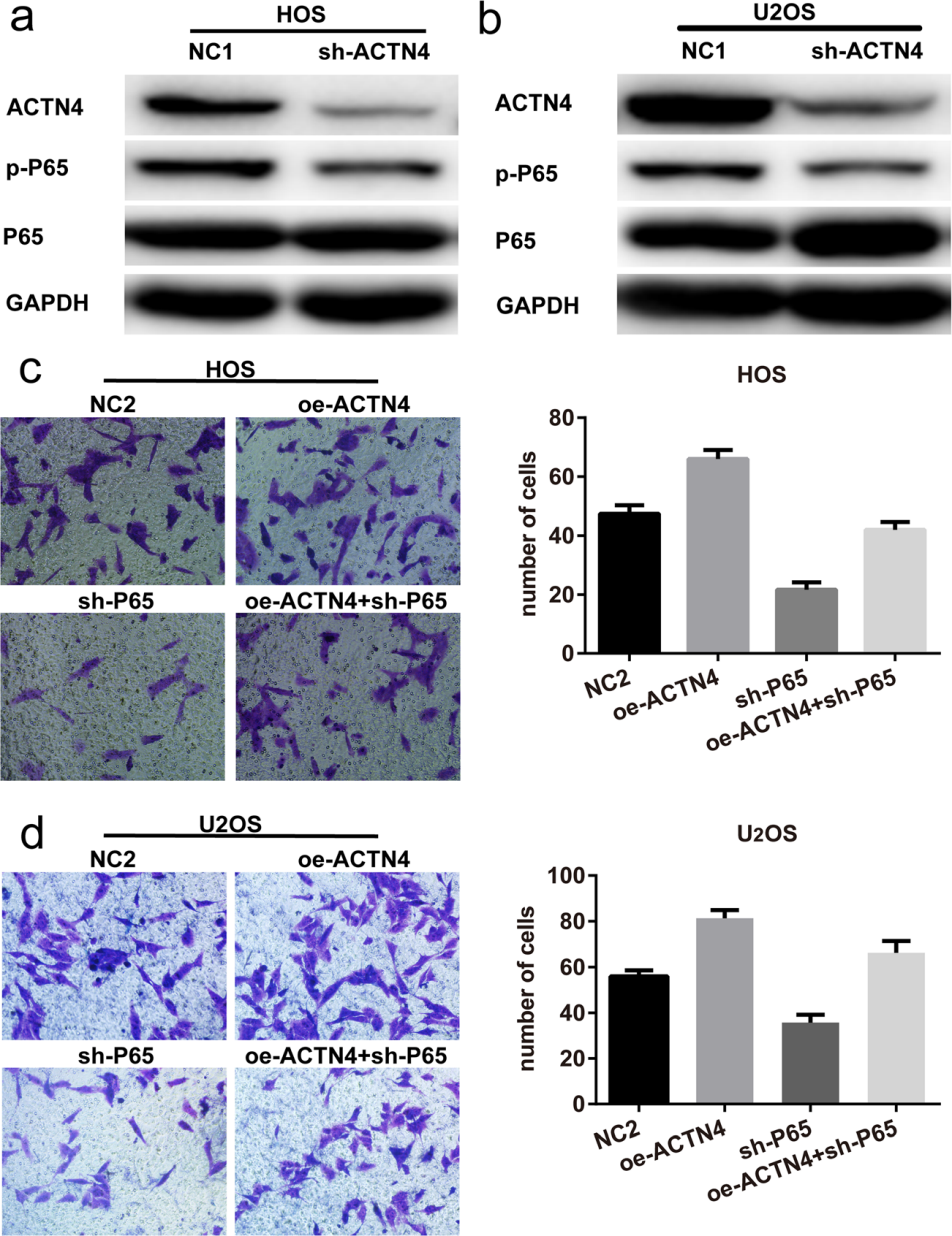
The mechanism of ACTN4 affecting the progression of OS. **a**, **b.** Western blots were used to detect the expression of phosphorylated P65 (p-P65) after ACTN4 interference. **c**, **d.** Changes in the invasive ability of HOS cells after ACTN4 upregulation or P65 downregulation. Data are shown as means ± SD

Phosphorylation of P65 is a measure of the activation of the NF-κB pathway, and we found that the expression of phosphorylated P65 (p-P65) was decreased when ACTN4 expression was reduced (Fig. 7a, b). Therefore, we hypothesized that ACTN4 may be involved in the activation of NF-KB in OS cells. We have previously demonstrated that overexpression of ACTN4 promotes the invasive ability of HOS cells. We further found that reducing P65 expression reversed the effect of ACTN4 on the invasive ability of HOS cells (Fig. 7c). We observed a similar trend in U_2_OS cells (Fig. 7d). Therefore, we speculate that ACTN4 may affect the invasive ability of OS through the NF-κB pathway.

## Discussion

The age of onset of OS is early and has a serious impact on the body and mind of patients. However, recurrence and metastasis of OS often lead to poor prognosis, which makes treatment of OS difficult. Therefore, there is an urgent need to find more effective treatments. Our study showed that ACTN4 promotes the proliferation, migration and invasion of OS cells in vitro. ACTN4 promotes the growth and metastasis of OS in nude mice. We further explored the possible mechanism of ACTN4 affecting the invasion of OS. In addition, our data suggests that ACTN4 enhances the invasive ability of OS through NF-κB pathway.

The role of ACTN4 in OS has rarely been reported. A recent study, however suggested that ACTN4 promotes the migration of OS cells [25]. Our study found that ACTN4 expression levels were significantly up-regulated in OS tissues and cell lines. We further confirmed that ACTN4 promotes the migration of OS cells using the scratch assay, which consistent with their findings. In addition, we observed that high expressions of ACTN4 promote the proliferation and migration of OS cells in vitro. We next validated the role of ACTN4 in OS in nude mice. At present, most of the OS xenograft models use subcutaneous or intravenous injections to observe tumor growth and metastasis. However, to better simulate the growth and metastasis of OS in the human body, we used orthotopic transplantation to inject OS into the bone marrow cavity of tibias in nude mice. Studies have shown that intramedullary injection can achieve tumor growth and metastasis with fewer cells [22]. Some researchers have also used this method to study the growth and metastasis of OS [26]. Finally, our results showed that ACTN4 promotes OS growth and lung metastasis in nude mice. Studies have shown that ACTN1 is highly expressed in breast cancer and is associated with its prognosis [27]. Our studies indicate that ACTN1 is also expressed in the HOS and U_2_OS cell lines. Therefore, ACTN1 may also have a certain effect on the function of OS cells, which is worthy of further investigation.

The activation of the NF-κB subunit P65 promotes the development of urothelial cancer and other tumors [28]. However, some researchers have found that P65 inhibits the proliferation of the lung cancer cell line H1299, and that overexpression of ACTN4 enhances this inhibitory effect [21]. Obviously, these two conclusions are in conflict with each other. Our results indicate that overexpression of ACTN4 promotes the activation of P65 in OS cells and ultimately enhances the invasion ability of OS cells through NF-κB pathway. Our conclusion is consistent with the former.

In summary, our results suggest that ACTN4 gene promotes the growth, migration, invasion and metastasis of OS. ACTN4 promotes the invasive ability of OS at least partially through the NF-κB pathway. These studies may provide new insights for the treatment of OS.
